# QSample: An Automated System for Rapid Monitoring of Quality Indicators in Proteomics Samples

**DOI:** 10.1021/acs.jproteome.5c00119

**Published:** 2025-08-19

**Authors:** Roger Olivella, Cristina Chiva, Marc Serret, Antoni Hermoso, Eva Borràs, Guadalupe Espadas, Julia Morales-Sanfrutos, Olga Pastor, Amanda Solé, Julia Ponomarenko, Eduard Sabidó

**Affiliations:** † Centre for Genomic Regulation (CRG), Dr Aiguader 88, Barcelona 08003, Spain; ‡ 16770 Universitat Pompeu Fabra (UPF), Barcelona 08003, Spain

**Keywords:** data analysis, nextflow, web application, mass spectrometry, automation

## Abstract

Mass spectrometry-based proteomics is an essential technique in contemporary biomedicine, offering quantitative, sensitive, and rapid analysis of proteomes. Recent advancements in mass spectrometry have enabled the acquisition of data from increasingly large-scale experiments, often conducted in core facilities and research infrastructures. While automated tools exist to assess instrument performance using predefined control samples, the analysis of experimental samples typically occurs postacquisition, which can delay decision-making and lead to potential data integrity issues. To address these challenges, we developed QSample, an open-source automated system for rapidly monitoring quality indicators in proteomics samples during data collection. QSample enhances the quality control framework by facilitating prompt actions and fast decision-making, ensuring that proteomics core facilities deliver data that adhere to best research practices.

## Graphical abstract

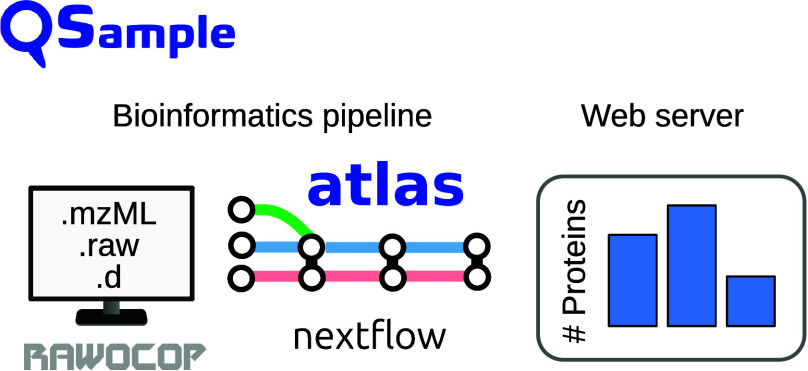

## Introduction

Mass spectrometry-based proteomics offers quantitative, sensitive, and rapid analysis of proteomes, making it one of the central molecular analysis techniques in contemporary biomedicine. Recent advancements in the stability, sensitivity, and speed of liquid chromatography–mass spectrometry instruments have facilitated the acquisition of data from increasingly large-scale experiments. Many of these experiments are performed in core facilities and research infrastructures that implement several types of quality control (QC) and quality assessment procedures^1,2^ and provide experience and expertise in many different proteomics applications.^3,4^ While several automated tools have proliferated to automatically assess longitudinal instrument performance using predefined control samples like QCloud and AutoQC,^5–9^ the analysis of experimental samples typically occurs only after the entire data set has been collected. This postacquisition approach allows for experiment-level analyses such as controlling the global false discovery rate in peptide and protein identification, performing peptide identity propagation (match between runs), normalization of peptide intensities, and batch correction.^10–12^ However, conducting automated sample analysis during the data set acquisition is advantageous for monitoring sample integrity and detecting potential data acquisition errors. This enables timely decisions such as repeating a sample injection or pausing the queue to address issues before valuable time and samples are lost. Unfortunately, the increased workload of evaluating samples during data collection can be tedious and create significant bottlenecks, as mass spectrometer operators often assess sample quality manually by inspecting individual raw files shortly after they are created. While manual inspection can identify many problems, some issues may go unnoticed at this initial stage, only to be detected later during data analysis. This might lead to delays, slow decision-making, and potential batch effects. To mitigate the late detection of problems in experimental samples, some tools have been developed that facilitate postacquisition data processing and enable the extraction of quality control metrics alongside data collection, particularly focusing on qualitative metrics or predefined internal standards, including Mascot Daemon, QC-ART, and Rapid QC-MS, among others.^13–16^

Here, we expanded this concept by developing QSample, an automated system for rapid monitoring of quality indicators in proteomics samples as soon as data are acquired in the mass spectrometer. QSample is an open-source system that operates on-premises, and it serves as an integral component of the proteomics quality control framework.^17^ QSample automatic analysis facilitates prompt actions and fast-decision making and, in sum, facilitates that proteomics core facilities acquire and deliver data conforming to the best research practices in the scientific community.

## Materials and Methods

QSample is composed of the Atlas pipeline (version 0.5.1), developed with Nextflow DSL2 syntax for proteomic data analysis, and a web server (version 0.4.3) developed with SpringBoot for data visualization. Together, these two parts provide researchers with automated workflows and a user-friendly interface that simplifies the interaction with data processing and the visualization of sample quality control metrics. With the combination of the Atlas pipeline and the QSample web server, QSample extracts a set of key quality control parameters (Table 1) from several predefined supported applications (Table 2). Each of these applications are associated with a default Atlas workflow, a default view, and default set of parameters (e.g., FDR <0.01, carbamidomethylation of cysteines as fixed modification, 7 ppm MS1 tolerance, etc.), which can be customized by the user in the corresponding configuration files for each of the supported tools (i.e., fragpipe-220.workflow, diann_192_bruker.cfg, diann_192.cfg, and openms.config found in the github repository).

**Table 1:** List of the Key Quality Indicators Available in QSample

name	description	related HUPO PSI controlled vocabulary	acquisition mode
number of protein groups	total of all protein groups present in the sample	count of identified proteins (MS:1002404)	DDA; DIA
number of peptides	total of all specific peptidoforms present in the sample	count of identified peptidoforms (MS:1003250)	DDA; DIA
sum of total TIC	total sum of all ion currents	total ion currents (MS:4000104)	DDA; DIA
peptides grouped by number of missed cleavages	count of peptidoforms with missed cleavages	table of missed cleavage counts (MS:4000180)	DDA; DIA
precursors grouped by charge	distribution of the number of peptide precursors with charge +2, +3 and +4	MS2 known precursor charges fractions (MS:4000063)	DDA; DIA
secondary reactions (% PSM)	secondary reactions expressed as a percentage of the total peptide spectral matches (PSM)	NA	DDA
polymer contaminants (% TIC)	polymer contaminants expressed as a percentage of total ion current (TIC) using the mzSniffer software tool	NA	DDA; DIA
correlation of protein abundances (Top 3)	correlation of protein abundances between samples from the same request	NA	DDA; DIA
number of sites modification	count of sites with a specific modification	NA	DDA
number of modified peptides	count of peptides containing at least one specified modification	NA	DDA

**Table 2:** List of the Predefined Applications and Their Default Associated Views and Parameters Available in QSample

default workflow tag	acquisition mode	workflow name	supported engines	default view	default variable modifications	default fixed modifications	default fragment mass tolerance	default fragment mass tolerance unit	default precursor mass tolerance	default precursor mass tolerance unit	default allowed missed cleavages	default FDR[Table-fn tbl2fn1]	secondary reactions available
MQ	DDA | DIA	Proteome label-free quantification	FragPipe, Comet, Mascot | DIANN	default	oxidation (M), acetyl (N-term)	carbamidomethyl (C)	0.5 (DDA) | auto (DIA)	Da (DDA) | ppm (DIA)	7 (DDA) | auto (DIA)	ppm	3 (DDA) | 1 (DIA)	0.01	Yes (FragPipe)
LA	DDA | DIA	Characterization of protein–protein interactions (AP-MS)	FragPipe, Comet, Mascot | DIANN	default	oxidation (M), acetyl (N-term)	carbamidomethyl (C)	0.5 (DDA) | auto (DIA)	Da (DDA) | ppm (DIA)	7 (DDA) | auto (DIA)	ppm	3 (DDA) | 1 (DIA)	0.01	Yes (FragPipe)
MG	DDA | DIA	Identification of a protein in a gel band	FragPipe, Comet, Mascot | DIANN	default	oxidation (M), acetyl (N-term)	carbamidomethyl (C)	0.5 (DDA) | auto (DIA)	Da (DDA) | ppm (DIA)	7 (DDA) | auto (DIA)	ppm	3 (DDA) | 1 (DIA)	0.01	Yes (FragPipe)
MC	DDA | DIA	Identification of an overexpressed protein	FragPipe, Comet, Mascot | DIANN	default	oxidation (M), acetyl (N-term)	carbamidomethyl (C)	0.5 (DDA) | auto (DIA)	Da (DDA) | ppm (DIA)	7 (DDA) | auto (DIA)	ppm	3 (DDA) | 1 (DIA)	0.01	Yes (FragPipe)
MP	DDA	Phosphoproteome label-free quantification	Comet, Mascot	modification	oxidation (M), acetyl (N-term), phospho (S), phospho (T), phospho (Y)	carbamidomethyl (C)	0.5	Da	7	ppm	3	0.05	No
MA	DDA	PTM (acetyl, methyl, phospho, ubiquitin) quantification of a purified protein	Comet, Mascot	modification	oxidation (M), acetyl (N-term), phospho (S), phospho (T), phospho (Y)	carbamidomethyl (C)	0.5	Da	7	ppm	3	0.05	No
LC	DDA	SILAC: checking incorporation	Comet, Mascot	modification	oxidation (M), acetyl (N-term), Label:13C(6)15N(4) (R), Label:13C(6)15N(2) (K)	carbamidomethyl (C)	0.5	Da	7	ppm	3	0.05	No
LP	DDA	SILAC: phosphoproteome quantification	Comet, Mascot	modification	oxidation (M), acetyl (N-term), Label:13C(6)15N(4) (R), Label:13C(6)15N(2) (K)	carbamidomethyl (C)	0.5	Da	7	ppm	3	0.05	No
LQ	DDA	SILAC: proteome quantification	Comet, Mascot	modification	oxidation (M), acetyl (N-term), Label:13C(6)15N(4) (R), Label:13C(6)15N(2) (K)	carbamidomethyl (C)	0.5	Da	7	ppm	3	0.05	No
LU	DDA	SILAC: ultra deep proteome quantification (fractionation)	Comet, Mascot	modification	oxidation (M), acetyl (N-term), Label:13C(6)15N(4) (R), Label:13C(6)15N(2) (K)	carbamidomethyl (C)	0.5	Da	7	ppm	3	0.05	No
MH	DDA	PTM quantification of histones	Mascot	histone	oxidation (M), acetyl (N-term), phenylisocyanate (N-term), propionyl (K), propionyl (protein N-term), dimethyl (K), trimethyl (K), acetyl (K), crotonaldehyde (K)	carbamidomethyl (C)	0.02	Da	7	ppm	3	0.05	No
MW	DIA	Targeted protein quantification (PRM)—A. method development	DIANN	default	oxidation (M), acetyl (N-term)	carbamidomethyl (C)	auto	ppm	auto	ppm	3	0.01	No
MT	DIA	Targeted protein quantification (PRM)—B. Measurement	DIANN	default	oxidation (M). acetyl (N-term)	carbamidomethyl (C)	auto	ppm	auto	ppm	3	0.01	No
LT	DDA	TMT: proteome quantification	Comet, Mascot	modification	oxidation (M), acetyl (N-term). TMT6plex (K). TMT6plex (N-term)	carbamidomethyl (C)	0.5	Da	7	ppm	3	0.05	No
LF	DDA	TMT: ultra deep proteome quantification (fractionation)	Comet, Mascot	modification	oxidation (M). acetyl (N-term), TMT6plex (K), TMT6plex (N-term)	carbamidomethyl (C)	0.5	Da	7	ppm	3	0.05	No
MK	DIA	Quantification using data independent acquisition (DIA)	DIANN	default	oxidation (M), acetyl (N-term)	carbamidomethyl (C)	auto	ppm	auto	ppm	1	0.01	No
BK	DIA	Quantification using data independent acquisition (DIA) – Bruker	DIANN	default	oxidation (M), acetyl (N-term)	carbamidomethyl (C)	auto	ppm	auto	ppm	1	0.01	No
LD	DDA | DIA	Chromatin-bound proteome	FragPipe, Comet, Mascot | DIANN	default	oxidation (M), acetyl (N-term)	carbamidomethyl (C)	0.5 (DDA) | auto (DIA)	Da (DDA) | ppm (DIA)	7 (DDA) | auto (DIA)	ppm	1	0.01	Yes (FragPipe)
ME	DIA	Proteome label-free quantification in exosomes	FragPipe, Comet, Mascot | DIANN	default	oxidation (M), acetyl (N-term)	carbamidomethyl (C)	0.5 (DDA) | auto (DIA)	Da (DDA) | ppm (DIA)	7 (DDA) | auto (DIA)	ppm	1	0.01	Yes (FragPipe)

* The FDR is set at 1% peptide and protein level for FragPipe and DIANN software, whereas it is set at 5% peptide level for Comet and Mascot.

The current Atlas code, together with a quick start guide and comprehensive documentation covering hardware and software requirements, installation, and configuration, is publicly accessible at https://github.com/proteomicsunitcrg/atlas/. Currently, the Atlas code supports third-party tools for data processing, including OpenMS (v3.1), FragPipe (v22), and DIANN (v1.9.2).

The QSample web server has been containerized by using the Docker Compose software, encapsulating all necessary dependencies to ensure high reproducibility across various computational infrastructures and environments. The QSample web server code, together with a quick start guide and comprehensive wiki documentation covering hardware and software requirements, installation, and configuration, is also publicly accessible at https://github.com/proteomicsunitcrg/qsample-server/.

## Results

In this study, we report the development of an open-source system for automatically monitoring quality indicators in proteomics experiments called QSample to enable users to quickly assess sample quality (Figure 1). Specifically, QSample is designed to support proteomics laboratories in the rapid assessment of different applications including the analysis of label-based and label-free proteome experiments that were acquired either with data-dependent or data-independent acquisition methods. For these applications, QSample is currently capable of extracting several key quality control parameters, including both identity-based and identity-free metrics, that substantially simplify their quality assessment (Table 1).

**Figure 1:**
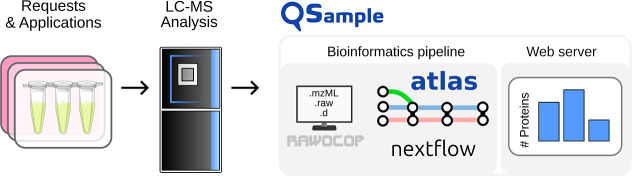
General structure of QSample, with a file transfer function (Rawocop), a bioinformatics pipeline (*Atlas*), and a web server for visualizing key quality indicators of samples associated with specific applications and grouped by request or experiment.

In QSample, each sample is connected to a specific experiment or *request* in the context of core facilities. These experiments or requests are then associated with particular proteomics *applications*. The requests determine the grouping of samples for display, while the applications represent predefined data analysis workflows associated with the experimental analyses conducted in the laboratory. The current version of QSample supports several predefined applications (Table 2), and for each them there is (i) an associated data analysis workflow, (ii) a database structure, and (iii) a customized view, which together calculate, store, and display the specific metrics established for each application. The specific parameters for data analysis are editable in a configuration file to align with the instrumentation used in each laboratory (e.g., mass accuracy, acquisition mode) and the particularities of each experimental workflow (e.g., alkylating reagent, expected modifications), among others. Immediately after acquisition, sample raw files are automatically uploaded into the QSample system and processed individually without any operator intervention in less than one hour (whole cell extract proteome sample, DIA, 30-SPD, Orbitrap Astral, 10 CPU, 16 GB). The resulting data are then presented on a web server for easy access and review.

QSample is designed to be nonprescriptive; it does not impose absolute thresholds for assessing individual samples, acknowledging that experiments in core facilities can vary widely based on sample type, taxonomy, and sample preparation, including fractionation and enrichment, among other factors. Instead, our tool contextualizes each metric to the other samples being acquired within the same experiment, facilitating a rapid evaluation of the homogeneity and stability of results within the experiment.

QSample is composed of the Atlas pipeline for proteomic data analysis, and a web server for data visualization. Together, these two parts provide researchers with automated workflows and a user-friendly interface that simplifies the interaction with the data processing and the visualization of sample quality control metrics. A demo version of the system is available at https://demo-qsample.crg.eu.

### Atlas Pipeline

Atlas is an automated proteomics data processing pipeline built on Nextflow.^18,19^ It is designed to support a variety of workflows, including data-dependent and data-independent acquisition modes as well as both label-free and label-based strategies (e.g., TMT, SILAC). The pipeline currently supports several search engines, such as Comet, Mascot, and MSFragger/FragPipe, along with DIA-NN for data-independent acquisition workflows (Figure 2).^13,20–23^ The current Atlas pipeline (v0.5.1) supports several predefined proteomics applications, each associated with a specific workflow and set of parameters (Table 2). While the pipeline comes with several predefined applications, users can customize the list of applications by creating new ones or modifying existing ones, and adjust their configuration settings (e.g., mass tolerances, variable and fixed modifications, search engine, etc.). Though some changes (e.g., new applications) might require a tight integration among the Atlas pipeline, the database, and the visualization layer, this flexibility enables researchers to tailor the Atlas pipeline to meet their specific experimental needs.

**Figure 2:**
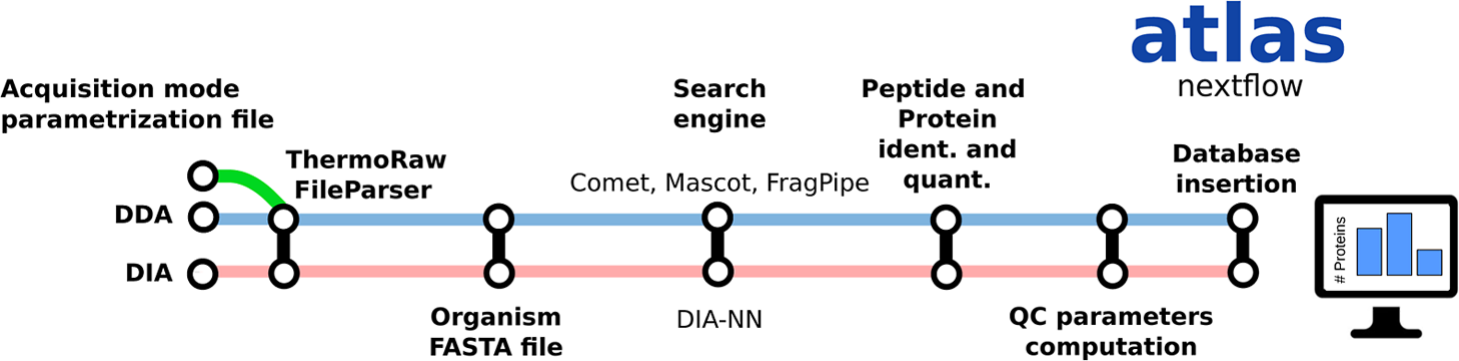
Scheme of the main bioinformatics pipelines supported by QSample, including data-dependent and data-independent acquisition file structures.

The pipeline accepts individual raw files—mzML or proprietary Thermo and Bruker raw files—accompanied by metadata, including experiment identifier codes (or request codes), taxonomy, and application details that need to be encoded within the file name or automatically appended by the input scripts from available metadata. This metadata determines how each file is analyzed: the *request identifier* code dictates how results are grouped on the display webpage (see QSample web server), the *application* specifies the parameters used during unattended data analysis, and the *taxonomy* determines the FASTA file utilized during analysis.

For Thermo RAW files, Rawocop software can be used to transfer and automatically annotate the raw files from XCalibur sequences as the first step into QSample.^24^ Rawocop is a lightweight and robust program written in.Net with a simple installation and configuration. Rawocop monitors a selected directory and evaluates its file content to automatically transfer new raw files, creating a folder structure based on the instrument type, serial number, and the current year and month into the destination path. With a compact size of just a few MB and a maximum RAM usage of 200 MB, Rawocop can be easily run on any mass spectrometer acquisition computer, supporting a wide range of new and old Windows versions. Individual raw files are thus automatically served on-the-fly by Rawocop from the acquisition computer as soon as the data acquisition is complete. Alternatively, files can also automatically be pushed into the pipeline incoming folder from other locations or storage solutions using *adhoc* scripts triggered postacquisition by the instrument control software (e.g., Xcalibur).

In terms of data output and analysis, the Atlas pipeline generates a range of quality control metrics, including the number of proteins and peptides identified, peptide charge distribution, number of miscleavages, total ion current, secondary reactions, and the percentage of polymer content, facilitated by integration with MZsniffer (Table 1).^25^ Additionally, the pipeline provides a list of the most abundant proteins and common protein contaminants per sample, with their estimated protein abundance.^26^ For applications focused on post-translational modifications and labeled workflows, it also tracks modified peptides and modification sites, providing a comprehensive view of the data to evaluate post-translational modification enrichment and labeling efficiencies (see the QSample web server for more details). The quality control metrics generated by the Atlas pipeline are stored in a MySQL database for visualization with the QSample web server or for automated export.

The Atlas pipeline ensures efficient processing times across various workflows and features testing and validation capabilities. These capabilities allow users to verify the pipeline’s functionality with different file types and generate concise output files for review. These features are complemented by high performance and scalability with configurable memory and CPU settings that optimize processing efficiency. The pipeline is compatible with both high-performance computing (HPC) clusters and desktop PCs, making it accessible for a wide range of research environments.

#### fastQSample Output Variant

The fastQSample is an Atlas output variant designed for users who require a streamlined and efficient method to access and analyze their mass spectrometry data without the need to install or run the full QSample web server. This variant enables complete decoupling between the analysis pipeline and the QSample web server, making it ideal for users looking for a direct way to process their requests and retrieve comprehensive sample quality control metrics. Upon execution, the fastQSample pipeline generates an mzQC and a tab-separated value (TSV) file as its output. These files are organized to provide all relevant information from each request identifier, ensuring that users have access to the corresponding quality control metrics in a single, easily accessible format. The TSV file includes entries for each specific file in the request along with detailed quality metrics. With these outputs, the fastQSample pipeline enhances interoperability of the QC metrics with other proteomics tools, and it ensures that users can easily import the output into a variety of software tools for further analysis, such as spreadsheet programs (e.g., Excel), statistical software (e.g., R), or custom scripts in programing languages like Python. This versatility makes these file formats particularly useful for users who need to integrate the data into their existing workflows or who prefer to perform custom downstream analyses.

### QSample Web Server

The QSample web server complements the Atlas pipeline by providing a user-friendly web application that facilitates data management and visualization of quality control parameters for the different samples, requests, and applications and simplifies the interaction with processed data. The QSample web server is developed using Spring Boot,^27^ the Angular framework,^28^ and the Plotly.js library,^29^ and it consists of a front-end web client, a back-end, and a database.

The current QSample web server (v0.4.3) offers a range of informative plots organized in different views tailored to the specific predefined application that are analyzed with the Atlas pipeline (Table 2, and Figure 3). The first plots are dedicated to the *Number of Protein Groups*, which displays the total count of protein groups detected in the sample, and the *Number of Peptides*, which indicates the total number of distinct peptidoforms defined by their unique amino acid sequence and modifications. Additionally, the *Number of Modification Sites* plot highlights the count of modified amino acids across all peptides, particularly useful for analyses involving phosphorylated residues in phosphoproteome applications or chemical and metabolic labeling experiments like SILAC and Tandem Mass Tags. The *Sum of Total Ion Current (TIC)* plot aggregates the total ion current for all spectra in a sample, providing insight into the overall signal intensity. Further, QSample includes plots that categorize peptidoforms by missed cleavages, showing the distribution of peptidoforms with 0, 1, 2, or 3 missed cleavages, and another that displays the distribution of peptide precursors based on their charge states (+2, +3, and +4). The *Polymer Contaminants* (%TIC) plot displays the percentage of total ion current attributed to polymer contaminants, including NP-40, Tween, Polysiloxane, Triton X-100, Polypropylene glycol, Polyethylene glycol, and their variants. Similarly, the *Secondary Reactions* plot, currently available only for data-dependent acquisition experiments analyzed with FragPipe, indicates the percentage of secondary reactions present in the sample. This includes common processes that occur during sample preparation such as amidation, ammonia loss, carbamylation, formylation, deamidation, oxidation, acetylation, unwanted carbamidomethylation, water loss, methylation, and isotopic peak errors, defined as mass offsets in MSFragger. Lastly, QSample offers a table displaying the five most intense proteins and common protein contaminants per sample, calculated based on the mean of the three most intense peptides, along with a correlation plot of protein abundances across samples. All of these visualizations provide a comprehensive overview of the proteomics data extracted by the Atlas pipeline, enabling in-depth analysis and interpretation. Although new metrics can be defined in the Atlas pipeline, integrating them into the QSample web server requires further configuration adjustments to ensure coordination among the Atlas pipeline, the database structure, and the visualization layer.

**Figure 3:**
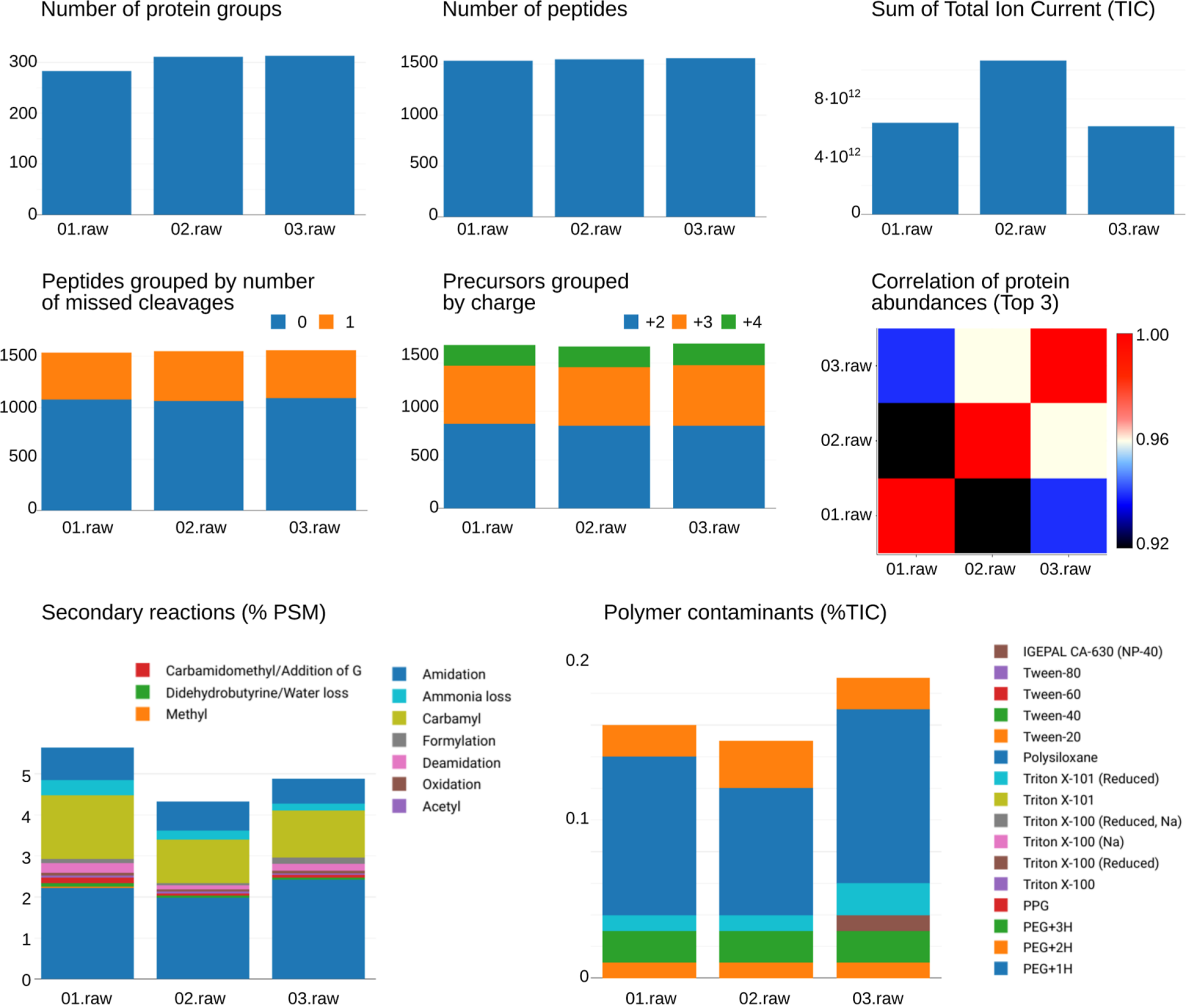
Main plots of the QSample web server automatically generated to display the key quality indicators of each sample within a request.

#### Wet Lab Module

The QSample web server also features a dedicated *Wet Lab Module* designed to automatically analyze external quality control samples (protein-level QC2) processed alongside experiments.^9,17^ These samples can be defined by each laboratory (e.g., *E. coli* or human whole cell extracts) and are intended to serve as positive controls to assess various aspects of the sample preparation process, such as digestion methods (in-solution, in-gel, in-filter), sample fractionation, and sample enrichment (e.g., phosphoenrichment). These external experimental controls are processed in the QSample system also as predefined applications (Table 2), but with a particular visualization layer in which samples are not organized by request but by week and batch of sample preparation. The module also offers the calculation of the mean and the standard deviation for each batch of quality control replicates, providing an overview of the quality and consistency of the sample preparation process over time (Figure S1A).

#### User and Request Management

Finally, the QSample server includes two additional modules for user and request management within the server.

The *User Management Module* of the QSample web server is designed to handle the QSample user accounts and their role within the system (*internal* and *lab manager*). The role of the QSample users determines their ability to create and modify new users and requests within the system, which are features enabled only for *lab manager* roles. The module is therefore only available for *lab managers* and it displays the list of all users, along with their details and roles, allowing for managing user permissions and adding and deleting users (Figure S1B). The integration of these features creates a robust user management system that supports both security and usability.

The QSample web server also integrates a *Requests Management Module* available to *lab managers’* roles that offers features for creating, filtering, and viewing existing requests (or experiments). Lab managers can filter requests based on status, application, request code, and creator and specify a date range to see requests created within a particular period (Figure S1C). The requests are displayed in a list format, with columns for request code, application, creator, request date, and status. During request creation, users with the *lab manager* role must provide a request code with a pattern and application name that match those defined in the configuration files. Requests also include the date and time of creation, laboratory name, user name, and organism taxonomy. Additionally, users can select the request status from a drop-down menu and have the option to add sample names. The interface facilitates the efficient management and organization of requests, enhancing the overall workflow within the QSample system.

## Conclusions

In conclusion, QSample is an automated, open-source system for the rapid monitoring of quality indicators in proteomics samples. By integrating the Atlas pipeline for data processing with a user-friendly web server, QSample streamlines the workflow for proteomics core facilities, enabling rapid sample quality assessment across various proteomics applications. Furthermore, the inclusion of dedicated modules facilitates data visualization, request and user management, and a comprehensive overview of sample preparation processes. Overall, QSample is an important part of the quality control framework by facilitating prompt actions and fast decision-making, ensuring that proteomics core facilities deliver data that adhere to best research practices.

## Supplementary Material



## Data Availability

All scripts and code presented in this manuscript is publicly available at the Github repositories https://github.com/proteomicsunitcrg/atlas/ and https://github.com/proteomicsunitcrg/qsample-server/.

## Notes

Screenshots of the QSample server modules on Wetlab (A), Request (B), and User management (C) (PDF)
