# Supplementary material for: QSample: An Automated System for Rapid Monitoring of Quality Indicators in Proteomics Samples
Source: J Proteome Res. 2025 Aug 19;24(9):4816–24. doi: 10.1021/acs.jproteome.5c00119 (PMC12418486; doi:10.1021/acs.jproteome.5c00119)

Supporting Information

Technical Note

QSample: an automated system for rapid monitoring quality indicators in proteomics samples

Roger Olivella[1,2,#]; Cristina Chiva[1,2,#]; Marc Serret[1,2]; Antoni Hermoso[1,2]; Eva Borràs[1,2]; Guadalupe Espadas[1,2]; Julia Morales-Sanfrutos[1,2]; Olga Pastor[1,2]; Amanda Solé[1,2]; Julia Ponomarenko[1,2]; Eduard Sabidó[1,2,*]

1. Centre for Genomic Regulation (CRG), Dr Aiguader 88, 08003 Barcelona, Spain

2. Universitat Pompeu Fabra (UPF), 08003 Barcelona, Spain

[#] Equal contribution

[*] Corresponding Author

Corresponding Author

Eduard Sabidó, Centre for Genomic Regulation (CRG), Dr Aiguader 88, 08003 Barcelona, Spain, Universitat Pompeu Fabra (UPF), 08003 Barcelona, Spain. Email: eduard.sabido@crg.eu

Supplementary Figure S1: Screenshots of the Qsample server modules on Wetlab (A), and Request (B) and User management (C).

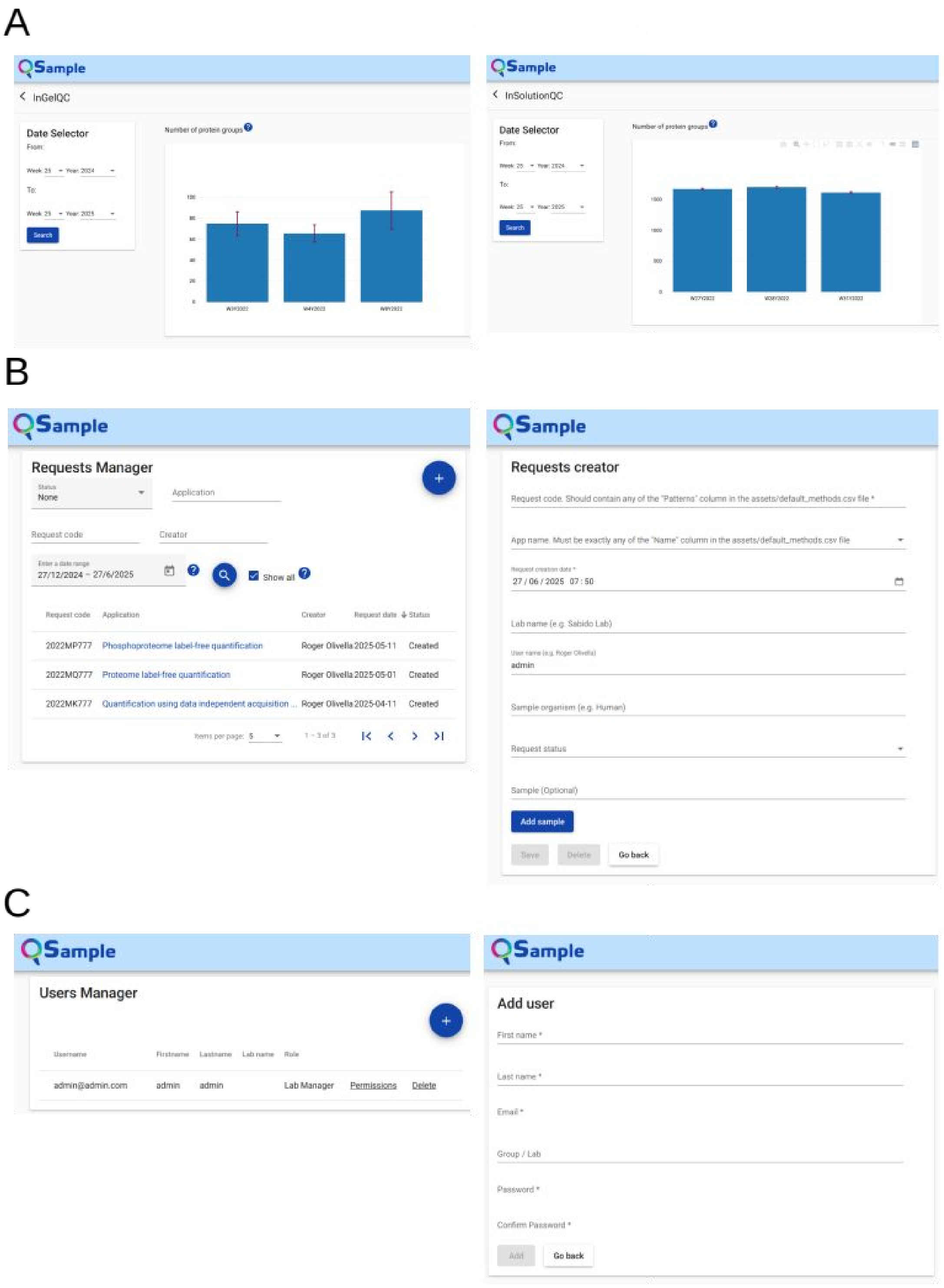

Supplement: Supplementary file 1 [file pr5c00119_si_001.pdf]
